# Efficient and reproducible experimental infections of rats with *Blastocystis* spp.

**DOI:** 10.1371/journal.pone.0207669

**Published:** 2018-11-19

**Authors:** Manon Defaye, Céline Nourrisson, Elodie Baudu, Ivan Warwzyniak, Virginie Bonnin, Mathilde Bonnet, Nicolas Barnich, Denis Ardid, Frédéric Delbac, Frédéric Antonio Carvalho, Philippe Poirier

**Affiliations:** 1 Université Clermont Auvergne, 3iHP, CNRS, Laboratoire Microorganismes: Génome et Environnement, Clermont-Ferrand, France; 2 Université Clermont Auvergne, 3iHP, Inserm U1107, NeuroDol, Clermont-Ferrand, France; 3 Université Clermont Auvergne, CHU, 3iHP, CNRS, Laboratoire Microorganismes: Génome et Environnement, Clermont-Ferrand, France; 4 Université Clermont Auvergne, 3iHP, Inserm U1071, USC INRA 2018, Microbes, Intestin, Inflammation et Susceptibilité de l'Hôte, Clermont-Ferrand, France; Universita degli Studi di Parma, ITALY

## Abstract

Although *Blastocystis* spp. infect probably more than 1 billion people worldwide, their clinical significance is still controversial and their pathophysiology remains poorly understood. In this study, we describe a protocol for an efficient and reproducible model of chronic infection in rats, laying the groundwork for future work to evaluate the pathogenic potential of this parasite. In our experimental conditions, we were unable to infect rats using vacuolar forms of an axenically cultivated ST4 isolate, but we successfully established chronic infections of 4 week-old rats after oral administration of both ST3 and ST4 purified cysts isolated from human stool samples. The infection protocol was also applied to 4 week-old C57BL/9, BALB/C and C3H mice, but any mouse was found to be infected by *Blastocystis*. Minimal cyst inoculum required for rat infection was higher with ST3 (10^5^) than with ST4 (10^2^). These results were confirmed by co-housing experiments highlighting a higher contagious potential of ST4 in rats compared to ST3. Finally, experiments mimicking fecal microbiota transfer from infected to healthy animals showed that *Blastocystis* spp. could easily infect a new host, even though its intestinal microbiota is not disturbed. In conclusion, our results provide a well-documented and robust rat model of *Blastocystis* chronic infection, reproducing “natural” infection. This model will be of great interest to study host parasite interactions and to better evaluate clinical significance of *Blastocystis*.

## Introduction

*Blastocystis* spp. are anaerobic enteric protist found in the intestinal tract of a wide range of animals, and are probably the most prevalent human parasites [1]. Four morphological stages of *Blastocystis* spp. have been described including amoeboid, granular, vacuolar and cystic stages. Both vacuolar and cystic forms are commonly found in human fecal samples and cyst is considered to be the infectious stage [1–3]. *Blastocystis* spp. have been classified into 17 subtypes (ST) based on nuclear small subunit (SSU) ribosomal RNA-encoding gene, the ST1 to ST9 and ST12 being recovered from human [4–7]. ST3 is the most frequent subtype in human, followed by ST1, ST2 and ST4 [5]. However, this distribution depends on geographic areas, and a higher prevalence of ST4 was reported from the north of Europe [8–10].

*Blastocystis* spp. infection occurs in both immunocompetent and immunocompromised hosts and recent studies highlighted higher prevalence of these parasites in patients with Irritable Bowel Syndrome (IBS) and colorectal cancer [11–14]. However, the involvement of *Blastocystis* spp. in human diseases is highly debated and well-designed epidemiological studies still need to be performed. Animal experimental models for *Blastocystis* infection have been purposed for proving Koch’s postulates, but few animal-based studies have been published so far, suggesting difficulties in the establishment and reproducibility of these models [15]. To date various animal models were used for *Blastocystis* infection as rodents (rats or mice), guinea pigs or chickens. Moreover, studies have shown that both vacuolar and cystic stages of different STs can efficiently infect animals [16–19]. The heterogeneity of infection methods with various animal models and/or different STs inoculation confirm that the establishment of a standardized model is necessary.

In this study, we evaluated several methods to inoculate *Blastocystis* spp. into laboratory animals (mice and rats) using different parasitic stages (vacuolar and cystic forms) isolated from *in vitro* cultures or from human or animal feces. Interestingly, we succeeded in the development of a reproducible model of chronic infection in juvenile and adult Wistar rats using purified cysts isolated from human stools.

## Materials and methods

### Animals

All animals were housed in animal biosafety level 2 (21–22°C, 12:12 h light-dark cycle) with access to food and water *ad libitum*. Three- and ten-week-old specific-pathogen-free (SPF) Wistar male rats and three-week-old SPF C57BL/9, BALB/C and C3H mice were purchased from Charles River Lab (Saint Germain Nuelles, France). All experiments were performed according to the ethical guidelines set out in the Guide for the Care and Use of Laboratory Animals and with approval of the “Comité d'Ethique pour l'Expérimentation Animale Auvergne" (C2E2A), the local ethics committee (Reference number: EU0116-3003). After experimental infections with *Blastocystis*, fecal samples were collected every week until sacrifice.

### *Blastocystis* ST4 axenic cultures

*Blastocystis* ST4 WR1 strain isolated from Wistar rats and axenized by Chen *et al*. [20] was cultured anaerobically in Iscove's Modified Dulbecco's Medium (IMDM) (Gibco, Life Technologies, Carlsbad, CA, USA) with 10% horse serum (PAA, Pasching, Austria) and 1% penicillin/streptomycin (Gibco, Life Technologies, Carlsbad, CA, USA) at 37°C. Vacuolar forms were collected from 48h-old cultures by centrifugation at 1000 g for 10 min and suspended in sterile PBS to reach a concentration of 10^7^ parasites per milliliter and were used for experimental infections.

### Oral administration of vacuolar forms isolated from axenic *Blastocystis* ST4 strain

In a first experiment, four-week-old Wistar male rats were orally inoculated with 10^7^ ST4 vacuolar forms per rat from *in vitro* axenic culture (n = 14). Eight rats were used to evaluate kinetic of ST4 route through the gastrointestinal tract and six rats were kept to monitor the progression of infection over time. To reduce gastric acidity, rats received 3 oral administrations every 2 hours of cimetidine (50 mg/kg, Mylan, Canonsburg, PA, USA) (n = 4) or 0.2 M sodium bicarbonate (Cooper, Melun, France) (n = 4) before oral inoculation with 10^7^ vacuolar forms of ST4 in a second experiment. Similarly as before, four rats were used to evaluate kinetic of ST4 route through the gastrointestinal tract and four rats were kept to monitor the progression of infection over time.

### Kinetic of ST4 route through the gastrointestinal tract of experimentally-infected rats

After oral administration of ST4, rats were euthanized at 3 (n = 2), 6 (n = 2), 12 (n = 2) or 24 h (n = 2) post-inoculation (PI). Stomach content was collected at 3 h PI, stomach and duodenum contents at 6 h PI, ileum and caecum contents at 12 h PI, and caecum, proximal and distal colon contents at 24 h PI.

For the experiments with animals pretreated with cimetidine or sodium bicarbonate, euthanasia of rats was done at 3 or 6 h (n = 2 for each time and condition). Stomach contents were collected at 3 h PI and caecum contents at 6 h PI.

### Intra-caecal inoculation of vacuolar forms isolated from axenic *Blastocystis* ST4

Four-week-old Wistar male rats were intra-caecally inoculated with 10^7^ ST4 vacuolar forms per rat from axenic culture (n = 6). Briefly, after 24 h of fasting, rats were anesthetized (Ketamine 75 mg/kg + Xylazine 10 mg/kg), followed by an abdomen incision, caecum externalization and injection of 10^7^ ST4 vacuolar forms. Then, caecum was repositioned and abdomen sutured.

### *Blastocystis* isolates collected from human stools

Four asymptomatic human carriers of *Blastocystis* ST2, ST3, ST4 and ST7 were identified at the medical laboratory of Parasitology of the Clermont-Ferrand teaching hospital (France). Both *Blastocystis* cysts and vacuolar forms were observed in stools for patients with ST2, ST3 and ST4, whereas only vacuolar forms were observed for the ST7 carrier. Fresh stools were collected for animal infections (approved by the research ethics committees of the Clermont-Ferrand Hospital, “Comité de Protection des Personnes Sud-Est 6”, France, agreement ref: 2014/CE29). Subtyping was performed by SSU rDNA sequencing as previously described [10,21]. Sequences were analyzed using the Basic Local Alignment Search Tool (BLAST; http://blast.ncbi.nlm.nih.gov/).

### Purification of cysts isolated from human stools

**Fig 1:**
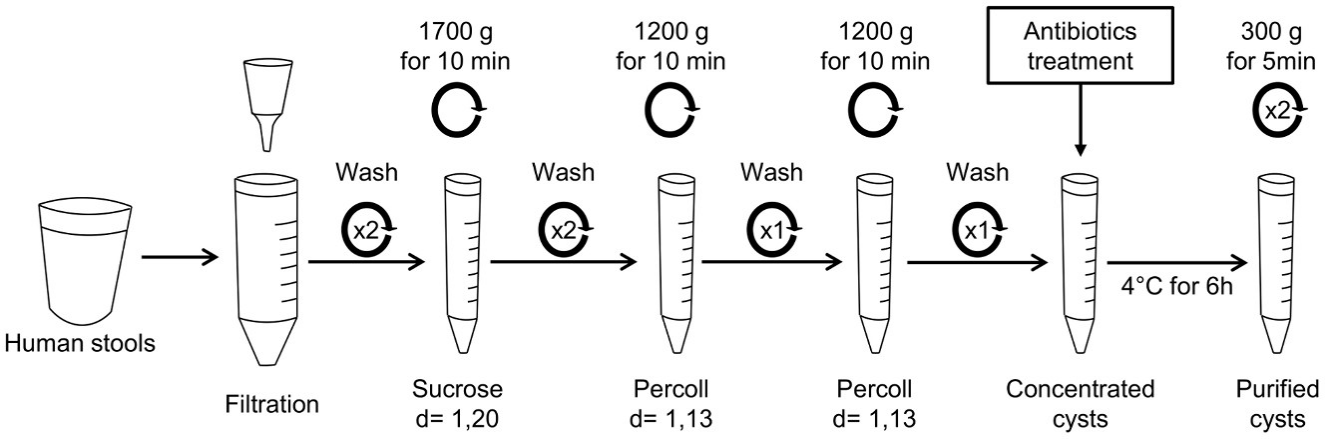
Method of cyst purification from human stools. About 50 g of stools were suspended in 200 ml of distilled water and filtered (1mm) to remove larger particles. After washing by centrifugation in distilled water (1700 g for 10 min), cysts were concentrated by sucrose gradient (d = 1.20) followed by two Percoll gradients (d = 1.13). Then, cysts were incubated with an antibiotic cocktail composed of Vancomycin, Amoxicilin-clavunalic acid and Cefotaxim to eliminate bacteria. After washing to remove antibiotics, purified cysts were suspended in sterile PBS and quantified. This method was adapted from Yoshikawa’s protocol [18].

Cysts were purified from human stools of ST2-, ST3- or ST4-infected patients (one patient for each subtype) by an optimized method (Fig 1) adapted from Yoshikawa’s protocol [18]. Almost 50 g of fresh stools were suspended in distilled water and larger debris were removed by filtration (funnel with filter disc 1mm, Theradiag, Marne La Vallee, France). The fecal suspension was washed twice in distilled water by centrifugation at 1700 g for 10 min. The supernatant was discarded and pellet was suspended in 40 ml of sucrose solution (d = 1.20) (Sigma-Aldrich, Saint-Louis, MO, USA) and was divided into 15 ml tubes. Then, 0.5 ml of distilled water was added in each tube followed by a centrifugation at 1700 g for 10 min. The interface between sucrose and distilled water was collected and suspended in distilled water. Two washes in distilled water were performed by centrifugation at 1700 g for 10 min. The pellet was suspended in 0.5 ml of distilled water and mixed with 7 ml Percoll solution (d = 1.13) (MP Biomedicals, Illkirch, France). Then, 6 ml of distilled water were added, followed by a centrifugation at 1200 g for 10 min. The interface between distilled water and Percoll solution was collected, adjusted to 15 ml with distilled water and centrifuged at 1700 g for 10 minutes. This step using Percoll gradients was performed twice. Pellets containing purified cysts were suspended in 0.5 ml of an antibiotic cocktail containing 0.01 g/L Vancomycin (Sandoz, Levallois-Perret, France), 0.1 g/L Amoxicillin-clavulanic acid (Sandoz, Levallois-Perret, France) and 0.1 g/L Cefotaxim (SteriMax, Oakville, ON, Canada) diluted in sterile PBS (Gibco, Life Technologies, Carlsbad, CA, USA) and incubated for 6 h at 4°C. Cysts were washed twice in sterile PBS by centrifugation at 300 g for 5 min and finally suspended in sterile PBS. Ten microliters of the suspension were plated on LB media and incubated at 37°C for 24 h in order to quantify the number of Colony Forming Units (CFU). Cysts were counted in Malassez chamber and suspension was diluted in sterile PBS to obtain concentrations of 10^2^, 10^3^,10^4^ or 10^5^ cysts per milliliter.

### Oral administration of purified cysts isolated from human stools

Wistar male rats (four or twelve-week-old) were orally inoculated with 10^2^/rat to 10^5^/rat ST2, ST3 or ST4 purified cysts isolated from human stools (n = 5 for each condition). *Blastocystis* isolated from rat feces after infection were subtyped by sequencing the SSU rRNA encoding gene as described above. For mice infections, 10^5^ ST4 purified cysts per animal were orally inoculated to four-week-old C57BL/6 (n = 3), BALB/C (n = 3) or C3H mice (n = 3).

### Co-housing experiments

Four-week-old Wistar male rats were orally inoculated with 10^5^ ST3 (n = 2) or ST4 (n = 2) purified cysts per animal, as described above. Four-weeks PI, these animals were co-housed for 6 weeks with eight-week-old non-infected rats (n = 4 per ST).

### Oral administration of rat feces containing *Blastocystis* cysts

Fresh feces from experimentally-infected rats, containing ST3 or ST4 cysts, were collected. Almost 300 mg of feces samples were pooled and suspended in 3 ml of sterile PBS. Then, eight-week-old rats (n = 3, for each ST) were orally inoculated with 10^5^ cysts.

### Fecal microbiota transplantation (FMT) using *Blastocystis*-positive human stools

Fresh stools from infected humans, containing ST2 or ST3 or ST4 cysts, or ST7 vacuolar forms, were collected. Almost 30 g of each fecal samples were suspended in 90 ml of sterile PBS. Moreover, almost 30 g of ST4 fecal sample were cryopreserved in 90 ml of PBS/glycerol 10% and stored at -80°C for two months. Four-week-old Wistar male rats were orally inoculated with 1 ml (corresponding to 300 mg of Human stools) of the suspension containing either ST2 (n = 3), ST3 (n = 3), ST4 with or without cryopreservation (n = 3 for each condition) cysts or ST7 (n = 3) vacuolar forms.

### Xenic cultures of *Blastocystis*-positive animal or human feces

Human or rat feces were cultured in Jone’s medium at 37°C in anaerobic chambers as previously described [22]. After 48 h of incubation, the culture was observed by standard light microscopy (x 400). Identification of vacuolar forms revealed the presence of viable parasites.

### Detection of *Blastocystis* by quantitative PCR (qPCR)

DNA was extracted from gastrointestinal tract contents or fecal samples of rats using the NucleoSpin Soil kit protocol (Macherey-Nagel SARL, Hoerdt, France). DNA was amplified with LC-FastStart DNA Master SYBR green kit (Roche Diagnostics, France) and specific primers BL18SPPF1 (5’-AGTAGTCATACGCTCGTCTCAAA-3’) and BL18SR2PP (5’-TCTTCGTTACCCGTTACTGC-3’) using a Rotor-Gene 6000 system (Corbett Life Science, France) as described by Poirier *et al* [10].

### Immunofluorescence labeling of *Blastocystis* cysts

Purified cyst smears were incubated with 5% milk in PBS for 1 h and washed in PBS/Triton-X100 0.1%. Then, cysts were incubated with anti-*Blastocystis* mouse polyclonal antibodies (diluted 1:200 in PBS) for 1 h at room temperature (RT), followed by three washes, and incubated with AlexaFluor 488-conjugated secondary antibody (anti-mouse IgG, 2 μg/ml in PBS) (Invitrogen Carlsbad, CA, USA) for 1 h at RT. Preparations were washed twice with PBS and stained with 4',6-diamidino-2-phenylindole (DAPI).

### Immunofluorescence staining of intestinal sections

Four weeks PI, ST4- or ST3-infected rats were euthanized and the intestine was prepared as “Swiss rolls” of 4 cm and fixed by incubating in PFA 4% during 24 h. Then, the rolls were transferred in sucrose 30% during 24–48 h at 4°C. Finally, the rolls were included in OCT compound (CellPath, Newton, United Kingdom). Sections of OCT-embedded intestine were incubated with 5% milk in PBS for 1 h, and then washed in 0.1% Triton-X100 in PBS. Anti-*Blastocystis* mouse polyclonal antibodies (diluted 1:200) were added for 1 h at RT, washed, and incubated further 1 h at RT with AlexaFluor 546-conjugated secondary antibody (diluted 1:1000) (anti-mouse IgG, Thermo Fisher Scientific, Waltham, MS, USA). Sections were washed and then incubated with fluorescein phalloidin (Thermo Fisher Scientific, Waltham, MS, USA) diluted 1:100 in PBS during 20 min. Finally, sections were washed and stained with DAPI. For each ST, six sections of duodenum, jejunum, ileum, caecum and colon from three rats were observed.

### Transmission electron microscopy (TEM)

Samples were washed in 0.2 M Sodium Cacodylate buffer and fixed overnight at 4°C with a mixture of 2% glutaraldehyde and 0,5% PAF in 0,2 M Sodium Cacodylate buffer. Specimens were then washed three-times in 0.2 M Sodium Cacodylate buffer, post-fixed 1 h with 1% OsO4 in 0.2 M Sodium Cacodylate buffer, and washed three-times (10 min) in 0.2 M Sodium Cacodylate buffer. Specimens were then dehydrated in a graded ethanol and acetone solution. Subsequently, they were infiltrated with acetone and EPON resin mixture (2:1) for 1 h, with acetone and EPON resin mixture (1:1) for 1 h, and with acetone and EPON resin mixture (1:2) for 1 h. Specimens were embedded in resin overnight at RT, and cured for 2 days in a 60°C oven. Thin sections (70 nm) were cut using a UC6 ultramicrotome (Leica, Wetzlar, Germany) and stained with uranyl acetate and lead citrate. Carbone was evaporated using CE6500 unit. Specimens sections were observed at 80 kV with a Hitachi H-7650 TEM and a camera Hamamatsu AMT40.

Electron microscopy preparations were all performed by the “Centre d’Imagerie Cellulaire Santé” (Clermont-Ferrand, France). All chemical products were from Electron Microscopy Science, and distributed in France by Delta Microscopies.

## Results

### Inoculation of rats with vacuolar forms of *in vitro*-cultivated *Blastocystis* ST4

In all our tested conditions, oral infections with vacuolar forms of *Blastocystis* ST4 axenic cultures failed (Table 1) even in animals pretreated with molecules which increase intragastric pH (cimetidine 50 mg/kg or sodium bicarbonate 0.2 M). To confirm the absence of parasites, we performed a follow-up of the parasite course through the intestinal tract of rats from 3 h to 24 h post-infection (PI) by xenic culture and qPCR. Even though high parasite loads were inoculated (10^7^ per animal), xenic culure and qPCR were all negative suggesting that vacuolar forms are not able to pass through the stomach alive (Table 2). After treatment with cimetidine or sodium bicarbonate, qPCR analysis revealed the presence of *Blastocystis* DNA in the caecum of rats 6 h PI but no viable parasitic form was observed after xenic cultures from collected caecum contents (Table 2). To avoid stomach passage, ST4 vacuolar forms were directly inoculated into the caecum of rats, but here again all animals remained uninfected (Table 1). These data suggest that vacuolar forms are not able to infect rats in our experimental conditions.

**Table 1:** Experimental infections of rats and mice with *Blastocystis* spp.

Stage	Stage treatment	Origin	Strain	Inoculation	Infectious dose	Animal (age)	Animal treatment	Xenic culture	qPCR	Results
Vacuolar forms	/	Axenic culture	ST4	Oral	10^7^	Wi4	/	-	-	Not infected
				Oral	10^7^	Wi4	Cimetidine 50 mg/kg	-	-	Not infected
				Oral	10^7^	Wi4	Sodium bicarbonate 0.2 M	-	-	Not infected
				Intra-caecal	10^7^	Wi4	/	-	/	Not infected
Cysts	Purified	Human stools	**ST2**	**Oral**	**10**^**5**^	**Wi4**	**/**	**+**	**/**	**Infected**
			**ST3**	**Oral**	**10**^**5**^	**Wi4**	**/**	**+**	**/**	**Infected**
				Oral	10^4^	Wi4	/	-	/	Not infected
				Oral	10^3^	Wi4	/	-	/	Not infected
				Oral	10^2^	Wi4	/	-	/	Not infected
			**ST4**	**Oral**	**10**^**5**^	**Wi4**	**/**	**+**	**/**	**Infected**
				**Oral**	**10**^**4**^	**Wi4**	**/**	**+**	**/**	**Infected**
				**Oral**	**10**^**3**^	**Wi4**	**/**	**+**	**/**	**Infected**
				**Oral**	**10**^**2**^	**Wi4**	**/**	**+**	**/**	**Infected**
				**Oral**	**10**^**5**^	**Wi12**	**/**	**+**	**/**	**Infected**
Cysts	Purified	Human stools	ST4	Oral	10^5^	C57BL/6 Mice	/	-	-	Not infected
						(4 week-old)				
						BALB/c Mice	/	-	-	Not infected
						(4 week-old)				
						C3H Mice	/	-	-	Not infected
						(4 week-old)				
Cysts	/	Human stools	**ST2**	**Oral**	**nd**	**Wi4**	**/**	**+**	**/**	**Infected**
	/		**ST3**	**Oral**	**nd**	**Wi4**	**/**	**+**	**/**	**Infected**
	With or without cryopreservation		**ST4**	**Oral**	**nd**	**Wi4**	**/**	**+**	**/**	**Infected**
Vacuolar forms	/	Human stools	ST7	Oral	nd	Wi4	/	-	/	Not infected
Cysts	/	Rat feces	**ST3**	Oral	10^5^	Wi4	/	-	/	Not infected
				**Co-housing**	**nd**	**Wi8**	**/**	+*	**/**	**Infected**
			**ST**4	**Oral**	10^5^	**Wi4**	**/**	**+**	**/**	**Infected**
				**Co-housing**	**nd**	**Wi8**	**/**	**+**	**/**	**Infected**

“/”: not carried out; “+”: positive result; “-“: negative result; “nd”: not determined;

“*”: only 1/4 naive rat became infected with ST3; Wi4: Wistar rats 4-week-old; Wi8: Wistar 8-week-old; Wi12: Wistar rats 12-week-old.

**Table 2:** Kinetic of *Blastocystis* ST4 route through the intestinal tract.

Animal treatment	PI Time	Part of the gastrointestinal tract	qPCR	Xenic culture
NA	3h	Stomach	-	-
NA	6h	Stomach	-	-
		Duodenum		
NA	12h	Ileum	-	-
		Caecum		
NA	24h	Caecum	-	-
		Proximal colon		
		Distal colon		
Cimetidine 50 mg/kg	3h	Stomach	+	-
	6h	Caecum	+	-
Sodium bicarbonate 0.2 M	3h	Stomach	+	-
	6h	Caecum	+	-

Four-week-old Wistar rats orally inoculated with 10^7^ vacuolar forms per animal. “NA”: not applied; “+”: positive result; “-“: negative result

### Infection of mice and rats with purified cysts isolated from human stool samples

**Fig 2:**
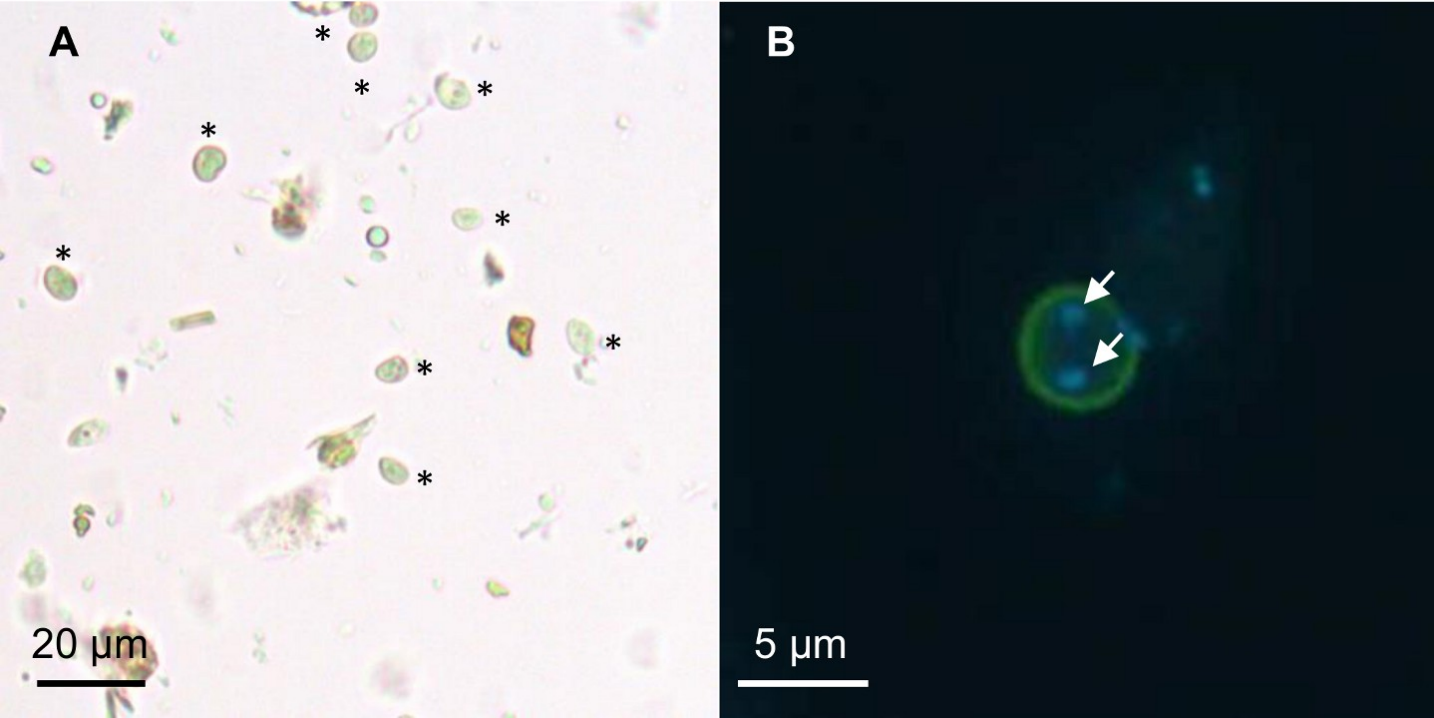
*Blastocystis* ST4 cysts purified from human stools. (A) Purified cysts from human stools observed by light microscopy. Numerous cysts can be observed (asterisk). More than 4 million of cysts were counted after purification from 50 g of human stool. Cysts size ranged from 2 to 7 μm. (B) Purified cysts from human stools observed by immunofluorescence after labeling with polyclonal anti-*Blastocystis* ST4 antibodies (Green) and DAPI staining (Blue). Cysts contained two to four nuclei (white arrows, two on this picture).

We optimized a purification protocol described previously by adding another Percoll gradient step and a treatment with an antibiotic cocktail (Fig 1). This protocol decreased drastically the number of bacteria below 250 CFUs inoculated per animal. From 50 g of human stool about 4 million cysts were purified for each subtype (Fig 2A). The size of purified cysts ranged from 2 to 7 μm and cysts were characterized by the presence of two or four nuclei (Fig 2B). Both juvenile (four-week-old) and adult (twelve-week-old) rats were successfully infected by oral inoculation of 10^5^ cysts of *Blastocystis* ST2, ST3 and ST4 per animal (Table 1). Cultures of feces collected 8 weeks after oral inoculation of cysts were positive for *Blastocystis*, confirming a chronic infection by the parasite.

We then evaluated the minimal inoculum for both ST3 and ST4 by using infectious dose ranging from 10^2^ to 10^5^ purified cysts per animal. Interestingly, rats were chronically infected with the lowest cyst inoculum of ST4 (10^2^), whereas higher inoculum of ST3 (10^5^) was required for the establishment of infection (Table 1).

During all experiments, only cystic forms were observed in feces from infected rats for both *Blastocystis* subtypes. Indeed excreted cysts in rat feces were characterized by light microscopy and immunofluorescence labeling using polyclonal antibodies. Cysts from ST3 and ST4 infected rats were similar in size to those isolated from human stools and were also characterized by the presence of two to four nuclei (S1 Fig).

The same infection protocol was further applied to 3 different genetic mice backgrounds, C57BL/6, BALB/c and C3H mice (Table 1). However, we were not able to obtain any infection, suggesting that immunocompetent mice could be resistant to infection by these ST4 *Blastocystis* strains.

### Transmission between animals by co-housing experiments or oral inoculation

Eight-week-old ST3 (n = 2) or ST4 (n = 2) infected rats were co-housed with eight-week-old naive rats (n = 4 per subtype) during six weeks. Infections were monitored by microscopy observation and xenic cultures of fecal samples. For ST4 co-housing experiments, all naive rats became infected only two weeks after the beginning of the experiment, whereas only 1/4 naive rat became infected with ST3, 6 weeks after the beginning of the experiment (Table 1).

Naive eight-week-old rats (n = 3 for each subtype) were also orally inoculated with feces from ST3 or ST4 infected rats. Only ST4-inoculated rats were infected (Table 1).

### Fecal microbiota transplantation from human to rats

Four-week-old rats were orally inoculated with fresh human stools containing *Blastocystis* ST2, ST3, ST4 or ST7, mimicking fecal microbiota transplantation. Both cystic and vacuolar forms were present in ST2, ST3 and ST4 human positive stools. In contrast, only vacuolar forms were observed in ST7-positive stools. Infection monitoring was done following FMT by xenic cultures of rat feces. Vacuolar forms were detected in culture feces from ST2, ST3 and ST4 infected rats but no viable parasite was identified in ST7-inoculated rat feces (Table 1). Interestingly, we also performed FMT from Human to rat after cryopreservation of *Blastocystis* ST4 stools, by following the recommended protocol for Human to Human FMT [23]. After 2 months at -80°C, stools were thaw out and transferred to naive rats. All animals were infected, suggesting that the procedure of cryopreservation use for Human to Human FMT also maintain *Blastocystis* alive.

### Colonization of the intestinal tract by *Blastocystis*

**Fig 3:**
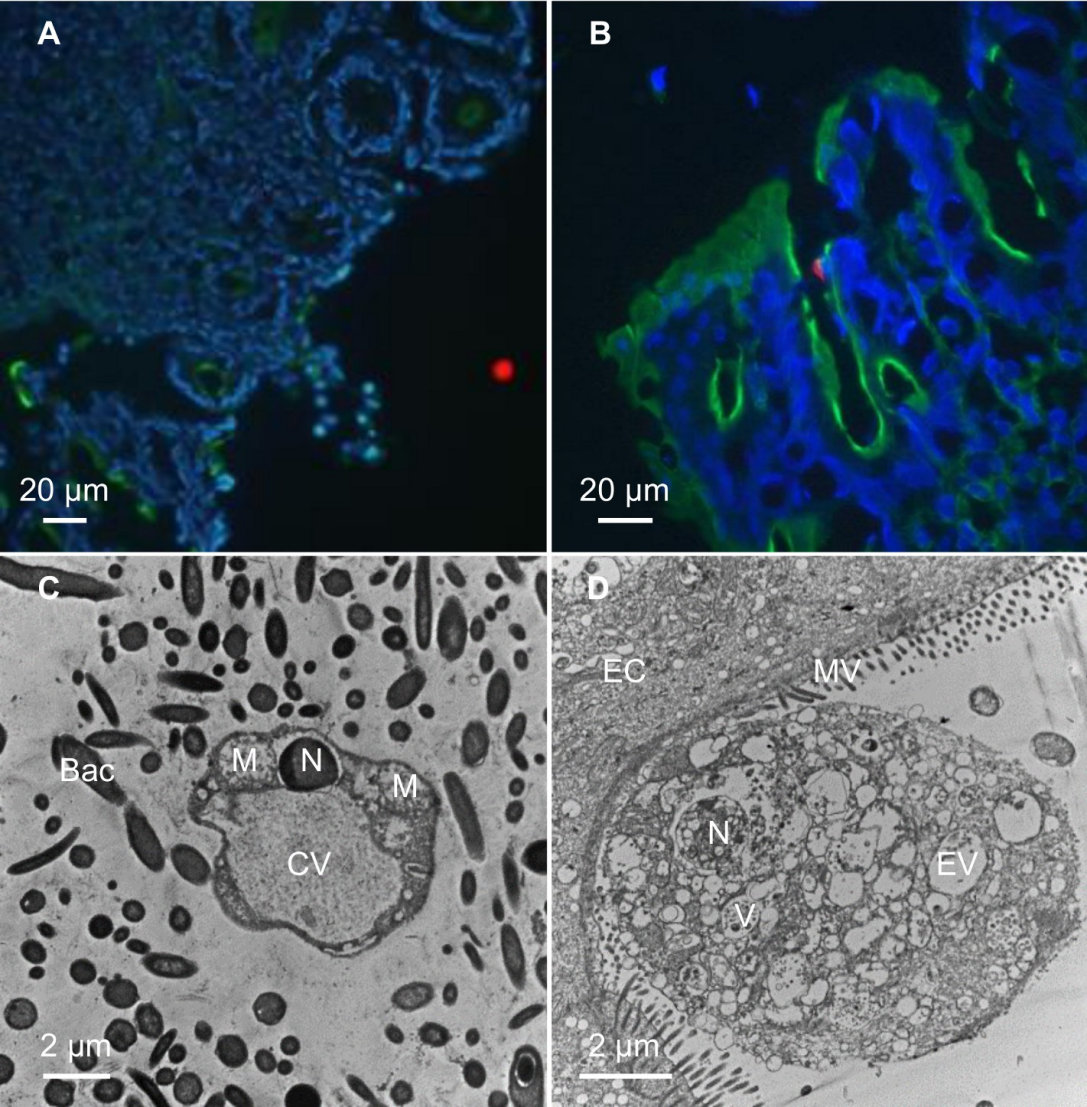
Colonic sections from *Blastocystis* ST4-infected rats. Colonic sections were stained with fluorescein phalloidin (Green), DAPI (Blue) and polyclonal anti-*Blastocystis* ST4 antibodies (Red). Vacuolar forms were detected in the intestinal lumen (A) and in close contact with the intestinal epithelium (B). Transmission electron micrographs of colonic sections from experimentally-infected rats *Blastocystis* vacuolar form surrounding by numerous bacteria localized in the intestinal lumen (C) and a granular form in close contact with an epithelial cell (D). M, mitochondrion-like organelle; N, nucleus, CV, central vacuole; EV, empty vacuole; V, vacuole; Bac, bacteria; EC, epithelial cell; MV, microvilli.

Both ST3- and ST4-infected rats by purified cysts from human stools were sacrificed four weeks PI. Full gastrointestinal tract was collected and “Swiss rolls” were performed on duodenum, jejunum, ileum, caecum, proximal and distal colon. *Blastocystis* vacuolar forms were observed from duodenum to distal colon for both ST3 and ST4 (S2 Fig). Immunofluorescence labeling revealed that parasites were mainly localized in the intestine lumen (Fig 3A), and sometime in close contact with epithelial cells (Fig 3B). No invasive form was observed. Transmission electron microscopy performed on colonic sections confirmed the presence of parasites in the lumen (Fig 3C) and in close contact with epithelial cells (Fig 3D).

## Discussion

Animal models are essential for a better understanding of the pathogenic potential of *Blastocystis* spp which still remains controversial. Rats (Wistar strain being the most common) [18,24–28], mice [16,17,29–31], guinea pigs [32] and chickens [25] were previously demonstrated as potential models of *Blastocystis* infection. However, to date [33] there is no standardized animal infection model for *Blastocystis*, suggesting difficulties in the establishment or reproducibility of these models.

In the present study, we aimed to provide a robust and well-described protocol to obtain efficient and reproducible experimental infection with *Blastocystis* spp. Four different human *Blastocystis* STs were used in our experiments, including ST3, the most common ST found in human, followed by ST2 and ST4, the latter reported to be highly prevalent in Europe [8–10], and ST7 whose whole genome was the first sequenced [33,34]. ST4 was also used because it is the most prevalent ST found in rodents and its whole genome was recently sequenced [35]. Parasites used in our study originated from axenic cultures (ST4-WR1, first isolated from a laboratory rat) [20] or were isolated from human stools by a highly efficient purification process (ST2, ST3 and ST4) adapted from Yoshikawa *et al* [18].

Based on the literature and the easiest models to handle in an animal facility, we focused on common rodent models with rats and mice. We were not able to infect rats with the axenic ST4 WR1 strain. In our *in vitro* culture conditions that strain only produced vacuolar forms. Then, failures in animal infections could be explained by sensitivity of vacuolar forms to gastric pH, but also to oxygen exposure during inoculum preparation [1,36]. Indeed, studies have shown that rat stomach pH ranged from 3.2–3.9 [37]. However, we also failed to infect animals with ST4 WR1 by increasing the intragastric pH or after intracaecal injection of parasites. Then, we hypothesized that long-term culture and/or axenization of vacuolar forms may have attenuated the infectious potential of the ST4 WR1 strain. Indeed, the maintenance of the parasites as *Trypanosoma cruzi*, *Entamoeba histolytica* or *Leishamania infantum* in laboratory cultures led to gene expression changes and decreased infectivity [38–41]. However, previous studies have reported successful infections of rats using axenic *Blastocystis* strains [16,17,19]. We cannot exclude that some axenic strains may conserve their infectious potential, or may produce cysts in axenic culture conditions. Indeed, cyst is considered to be the main infectious stage, responsible for feco-oral contamination between hosts [18,24,25,27,28,42]. Different protocols have been described for cyst isolation from human stool samples [17,18]. We optimized Yoshikawa’s protocol in order to decrease cyst-associated bacteria [18]. Then, an additional Percoll step was performed and purified cysts were incubated with a wide spectrum antibiotic cocktail targeting both aerobic and anaerobic bacteria. Moreover, successful infections following this treatment demonstrated low impact of this antibiotic treatment on *Blastocystis* cyst viability.

We were able to infect rats with ST2, ST3 and ST4 cysts by using high parasite load (10^5^ cysts/animal). Infected animals were followed as long as 8 weeks post-infection. Animals excreted *Blastocystis* cysts until the end of experiments, confirming the establishment of a chronic infection in our model.

Our results reinforced that ST4 infection failures were not related to a resistance mechanism, but more likely to the stage used or long-term *in vitro* cultivation. The susceptibility of animals to *Blastocystis* was reported to be age-dependent by Moe *et al*., but these authors didn’t mentioned the *Blastocystis* ST used in their experiments [17]. In our study, we were also able to infect twelve-old-week rats with *Blastocystis* ST4 cysts, suggesting that at least for this ST, age is not a limiting factor. However, the effects of aging on susceptibility to ST3 infection remain to be investigated. Interestingly, rats were infected with as low as 10^2^ ST4 cysts per animal, whereas the minimal inoculum dose required for infection with ST3 reached 10^5^ cysts suggesting host adaptation. Transmission between animal by co-housing experiments and oral inoculations confirm our hypothesis. Transmission between animal by co-housing experiments and oral inoculations confirm our hypothesis. These results may explain why ST4 is the more prevalent than ST3 in rodents [43]. However, oral inoculation with less than 10^2^ ST4 cysts per rat remains to be investigated, but a previous work has shown that infection efficiency with 10 cysts of ST4 RN94-9 or ST4 NIH:1295:1 strains varies between 20–100% [18].

Our results suggest that even though host barrier is not critical for *Blastocystis* infection, some STs are more adapted to particular hosts. Then, we applied our purification protocol to infect 3 strains of mice. The first one was juvenile BALB/c mice that have been shown to be more susceptible to parasite infections [44,45] such as *Blastocystis* [17,19]. We also used C3H mice that have been described to be susceptible to *Entamoeba histolytica* [46] and *Giardia intestinalis* [47]. Finally, C57BL/6 mice, being the most used genetic background for transgenic mice, were also used. However, we were not able to infect mice even with high ST4 cyst inoculum (10^5^/animal), suggesting that mice would be resistant to the ST4 strain used in our study. A recent study has shown that the Dextran Sodium Sulfate (DSS) treatment result in biophysical changes in mucus layer (increased penetrability to microorganism), increasing susceptibility of mice to *Blastocystis* ST7 colonization for at least 3 days after intra-ceacal injection [48]. These results suggest that mucus layer play in important role in the resistance of mice for *Blastocystis* persistence.

Immunofluorescence labelling revealed the presence of the parasites (ST4 or ST3) all along the intestinal tract of infected animals. *Blastocystis* were detected in the intestinal lumen and in close contact with epithelial cells. Granular forms presenting empty vacuoles or vacuoles containing electron-dense granules as described previously by Tan [1], were observed in direct contact with epithelial cells.

Moreover, FMT experiments highlighted the capacity of *Blastocystis* to overcome barrier microflora and infect a new host, even though its microbiota is not altered. We also demonstrated that cryopreservation procedure used for the stool storage before Human to Human FMT keep *Blastocystis* alive and able to infect a new host [23]. These data support the recent recommendations for the screening of fecal donors by confirming the ability of *Blastocystis* to be directly transmitted through stools [23].

In conclusion, our work provides a well-documented and reproducible animal model of *Blastocystis* chronic infection, reproducing “natural” infection. This model will be of great interest to decipher the host-parasite-microbiota interactions, and to better evaluate clinical significance of *Blastocystis*.

## Supporting information

S1 Fig *Blastocystis* ST4 cysts from experimentally infected rats. (**A**) Cysts (red arrow) from rats observed by light microscopy. As purified cysts from human stools, size ranged from 2 to 7 μm. (**B)** Cysts from rats observed by immunofluorescence after labeling with mouse polyclonal anti-*Blastocystis* ST4 antibodies (Green) and DAPI staining (Blue). Cysts contained two to four nuclei (white arrows, two on this picture).
(TIF)

S2 Fig Intestinal sections from *Blastocystis* ST4-infected rats. Sections of the intestinal tract were stained with fluorescein phalloidin (Green), DAPI (Blue) and mouse polyclonal anti-*Blastocystis* ST4 antibodies (Red). Parasites were detected in small intestine (**A**), in caecum (**B**) and colon (**C**) in the lumen or in close contact with the intestinal epithelium.
(TIF)
