# Supplementary figures and images for: Efficient and reproducible experimental infections of rats with Blastocystis spp
Source: PLoS One. 2018 Nov 19;13(11):e0207669. doi: 10.1371/journal.pone.0207669 (PMC6242359; doi:10.1371/journal.pone.0207669)

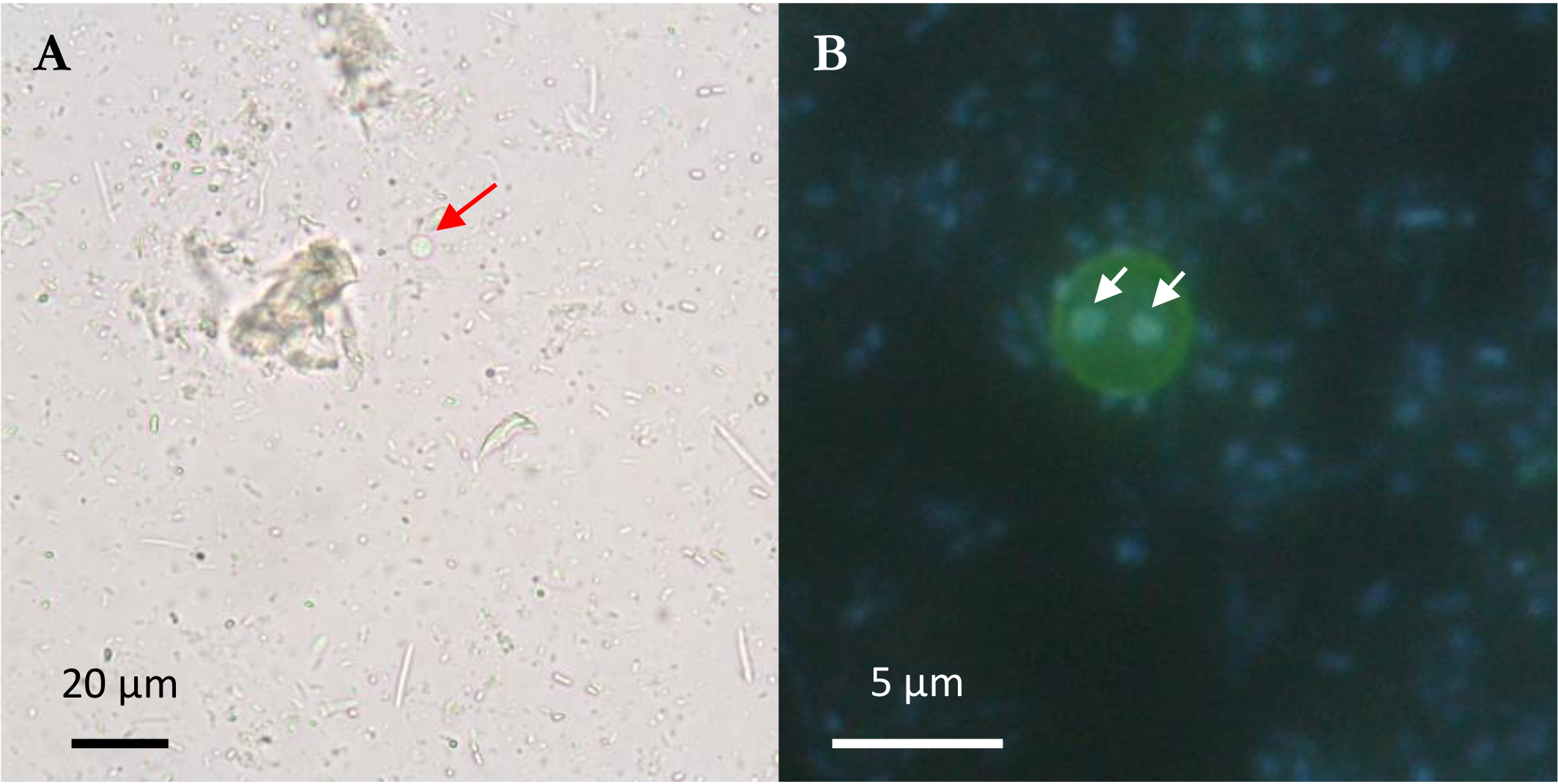

Supplement: S1 Fig — (A) Cysts (red arrow) from rats observed by light microscopy. As purified cysts from human stools, size ranged from 2 to 7 μm. (B) Cysts from rats observed by immunofluorescence after labeling with mouse polyclonal anti-Blastocystis ST4 antibodies (Green) and DAPI staining (Blue). Cysts contained two to four nuclei (white arrows, two on this picture). (TIF) [file pone.0207669.s001.tif]

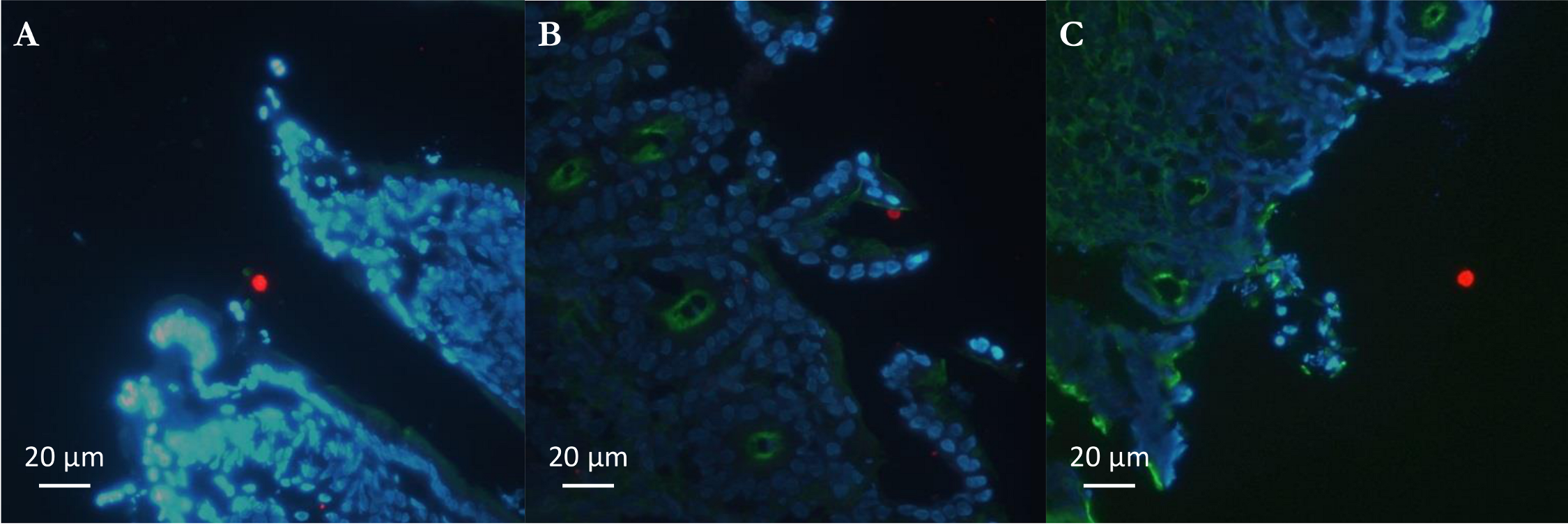

Supplement: S2 Fig — Sections of the intestinal tract were stained with fluorescein phalloidin (Green), DAPI (Blue) and mouse polyclonal anti-Blastocystis ST4 antibodies (Red). Parasites were detected in small intestine (A), in caecum (B) and colon (C) in the lumen or in close contact with the intestinal epithelium. (TIF) [file pone.0207669.s002.tif]
